# Ethical Considerations in Post-Mortem Sperm Retrieval: A Comprehensive Review

**DOI:** 10.5935/1518-0557.20240022

**Published:** 2024

**Authors:** Raghvendra Kumar Vidua, Nimisha Dubey, Tanu Budholia, Ashwani Tandon, Arneet Arora, Mrinal Patnaik

**Affiliations:** 1 Department of Forensic Medicine & Toxicology - AIIMS Bhopal, Bhopal, India

**Keywords:** postmortem sperm retrieval, ethics in PMSR, postmortem parenthood, PMSR Act and guidelines

## Abstract

This comprehensive review delves into the moral and ethical dilemmas surrounding post-mortem sperm retrieval (PMSR) and its implications for creating new individuals. The paper examines the challenges posed by unusual requests for sperm retrieval from the deceased’s widow and parents, as well as the broader socio-ethical considerations associated with PMSR. These requests have often been denied due to the absence of established laws and guidelines governing posthumous sperm retrieval and subsequent births, which were once deemed impossible. While some countries have implemented institutional policies to regulate its use to some extent, there remains a lack of standardized rules and procedures for the collection and retrieval of sperm after death. It is essential to introduce institutional guidelines to facilitate requests for assisted reproductive technology (ART) following successful sperm retrieval. Additionally, the development of PMSR legislation is necessary to ensure a proper balance between the moral rights and fundamental rights of the deceased, their family, and any current or future offspring, while providing adequate protection for all parties involved.

## INTRODUCTION

The advancement of technologies in various fields, particularly in medical sciences, brings about ethical issues that require careful consideration. Bioethics plays a crucial role in addressing these ethical concerns, providing a framework for moral deliberation in society and guiding medical norms and laws. Reproductive technologies, in particular, demand a special focus on bioethics and legal regulations. Within the realm of reproductive ethics, posthumous sperm retrieval techniques serve as reminders of the ethical challenges involved.

The history of post-mortem sperm retrieval (PMSR) dates back to 1980 when the first reported case was documented by Rothman (1980), involving a brain-dead man who had survived a motor vehicle accident. Subsequently, in 1994, a notable case in Florida emerged where a newlywed man tragically died in a car accident, and his wife expressed her desire to have his sperm frozen (Flores, 1994). Ohl *et al*. (1996) reported six cases, including requests for sperm retrieval from patients in a persistent vegetative state, comatose patients, and brain-dead patients.

In the context of posthumous sperm retrieval, not only are bioethical issues at stake, but social factors can also complicate the implementation of such practices, especially in countries like India. Physicians often acknowledge the potential difficulties faced by the widow of the deceased in making decisions regarding posthumous sperm retrieval. Social and familial pressures can easily influence the situation, making it a challenging time for the widow. Hasty or incorrect decisions made by the widow may lead to future complications.

In several developing countries, including India, the absence of guidelines for conducting ethical post-mortem sperm retrieval (PMSR) practices remains a significant challenge. Family members of the deceased often adhere to their cultural beliefs and are reluctant to embrace these novel techniques. In such situations, the prior consent of the deceased and his wife assumes great significance in facilitating assisted reproductive technology (ART) procedures. However, the lack of PMSR guidelines and related legislation in these countries has rendered such requests ethically unjustifiable, leading to their rejection in the past (Samal, 2018; ABP News Bureau, 2016).

## INTERNATIONAL STATUS OF PMSR

Different countries have varying stances on the use of posthumously retrieved sperm for reproductive purposes. France, Australia, Canada, Germany, Sweden, and some other nations do not support the use of posthumously retrieved sperm to create a child. However, in Western Asian countries like Israel, sperm harvesting is allowed even without the presumed consent of the deceased.

Arthur Caplan, a bioethics professor at New York University’s Medical Center, has emphasized the importance of maintaining certain principles to protect the autonomy of patients, whether they are alive or deceased. According to Caplan, the consent of the patient, their proxy, or statutory authority should hold the right to decision-making in post-mortem cases. However, in situations where the retrieval of viable sperm needs to be performed within a specific time frame, such as 24 to 36 hours (approximately 1 and a half days) after death, the concept of “presumed consent” can be considered. These principles proposed by Caplan aim to strike a balance between respecting the autonomy and wishes of the patient while also addressing time-sensitive situations where immediate action is required for successful post-mortem sperm retrieval.

The Weill Cornell Medical (WCM) New York Hospital has developed a comprehensive guideline for posthumous sperm retrieval (PMSR). This guideline was created by a panel of experts in various fields, including legal, ethical, medical, psychological, administrative, and lay experts. The purpose of this guideline is to address the various issues associated with PMSR and provide a framework for appropriate decision-making.

The WCM guideline places emphasis on several key aspects, including obtaining proper consent, considering the health condition of the deceased (PMSR candidate), determining the use of the retrieved sperm, and addressing the subsequent treatment and consequences of assisted reproduction. It specifies that requests for PMSR will only be accepted if made by the decedent’s wife, excluding other family members (Legislative Department, Ministry of Law and Justice, Government of India, 1994).

## POSTMORTEM SPERM RETRIEVAL: AN UNFITTING PRACTICE IN INDIA

Postmortem sperm retrieval remains a contentious and unfitting practice in countries like India. Despite the process of modernization, cultural and religious beliefs still hold significant influence in society. Some individuals view postmortem sperm retrieval as morally acceptable, while others consider it sinful and offensive to their religious sentiments, as it involves retrieving sperm from a deceased body. In India, there is currently a lack of specific laws that regulate post-mortem sperm retrieval, leading to potential legal restrictions in this area and the absence of specific laws poses challenges.

Ethical, legal, and medical guidelines specifically addressing post-mortem sperm retrieval (PMSR) in India are currently lacking. The existing guidelines, such as the ART (Regulation) Rule of 2010 drafted by the Indian Council of Medical Research (ICMR) (Ministry of Health and Family Welfare, 2010), focus on assisted reproductive technologies (ART) but do not mention PMSR or posthumous reproduction. While Schedule-1 Part 4, Section 4.6.2 of the ART Rule explains the intracytoplasmic sperm injection (ICSI) procedure using surgically extracted sperm in certain conditions, such as congenital absence of vas deferens, obstructed and non-obstructed azoospermia, anejaculation, and retrograde ejaculation, its application to posthumous insemination is not specifically mentioned.

Organ donations in India are considered a good practice and legal under the Transplantation of Human Organ Act (THOA), 1994 (Legislative Department, Ministry of Law and Justice, Government of India, 1994). The concept of permanent cessation of brain functions, i.e., “brain death” is completely legal in India. A medical practitioner may perform the further procedure of organ donation after the consent of the family (Herrington & Parker, 2019). But when we talk about sperm retrieval and donation practices from a dead body, it is still considered taboo. Because of no guiding principle, most of the time families deny giving consent as they believe that such things are illegal and ethically incorrect. The following issues are considered hurdles in the legitimacy of this procedure:

1. Lack of Clear Guidelines
2. Taboo and Cultural Beliefs
3. Inadequate Awareness
4. Consent of Family Members
5. Privacy and Confidentiality

## DISCUSSION

The issue of presumed or prior consent of the deceased is an important ethical consideration in post-mortem sperm retrieval. The guidelines established by Cornell emphasize that if explicit consent was not provided by the deceased or their family members, it raises ethical questions about proceeding with the procedure. In the context of consent, the wife of the deceased should be considered the primary source for determining the husband’s intention to procreate.

In a different study, it was found that family members of potential posthumous donors often refused to provide consent for sperm retrieval due to concerns about the thoughts and intentions of the deceased and his spouse. The past expressed interest of the deceased in becoming a parent raises the concept of “presumed or prior consent”. This implies that if there is a clear indication of the deceased’s desire to have children, their consent can be presumed in the absence of explicit documentation. Informed consent is a crucial ethical consideration in post-mortem sperm retrieval (PMSR). The consent of the deceased individual is required, and this can be obtained prior to their death or from a designated decision-maker, such as their partner or another family member. Respecting the autonomy and wishes of the deceased person after their death is essential in these cases.

Brian M. Cummings and John J. Paris address this issue in their article (Cummings & Paris, 2020), focusing on the choice and presumed consent of the deceased. They argue that without any consent, whether the person is alive or dead, no physician should perform any procedure on their body. The concept of presumed consent has been considered in cases of organ donation, where individuals express their wish not to donate organs after death. In the context of post-mortem sperm retrieval, the family should have the option of a “hard opt-out,” where consultation with the family was not conducted, or a “soft opt-out,” where the family is consulted, and their wishes are taken into consideration for further processing.

The consideration of presumed or prior consent provides a framework for addressing ethical concerns when explicit consent is not available, while still respecting the autonomy and intentions of the deceased individual. It offers options for the family’s involvement in decision-making, ensuring their perspectives are acknowledged and valued. Practices like post-mortem sperm retrieval (PMSR) can benefit from a comprehensive understanding of the procedure and the underlying need for its performance. This understanding should be accompanied by obtaining the informed consent of the widow and family members of the deceased individual. As highlighted by Sikary *et al*. (2016) in their review article, there is a growing awareness among people, facilitated by easy access to online healthcare information. In light of this, it becomes imperative to engage in thoughtful deliberations considering the overall benefit to society when addressing practices like postmortem sperm retrieval (Weill Cornell Medicine, 2017).

A study conducted by Shefi *et al*. (2006) indicated that sperm retrieval can be performed within 36 hours after death, as long as the reproductive organs remain undamaged. This suggests a time frame within which post-mortem sperm retrieval can be ethically justifiable, provided the necessary conditions are met. In a separate study by Strong (2006), the importance of a man’s prior or inferred consent for ethical justification of post-mortem sperm retrieval during a persistent vegetative state (PVS) and after death was highlighted. The study emphasized that the thoughts and wishes of the patient or survivor may differ from those reported by family members after the patient’s death.

Cummings & Paris (2020) published an article that sheds light on the ethical issues surrounding PMSR, particularly in cases where the spouse of the deceased is not present. The article discusses a specific case where the parents of a 21-year-old deceased individual requested court authorization to retrieve their son’s sperm.

To provide a solid foundation for PMSR, it is essential to either include specific principles and guidelines within existing rules or develop new regulations specifically addressing this practice. These guidelines should address the ethical, legal, and medical considerations unique to PMSR, ensuring clarity and providing a framework for healthcare professionals and individuals involved in these procedures. This would promote the ethical and responsible practice of PMSR and ensure the protection of all parties involved (Husain, 2000).

Religious and cultural beliefs play a significant role in shaping attitudes towards post-mortem sperm retrieval. In India, a diverse country with various religious and cultural beliefs, there are differing views on the practice. Some religious perspectives may consider post-mortem sperm retrieval as interference with the deceased body, raising ethical concerns about respect for the dead and the preservation of dignity.

The acceptance and understanding of post-mortem sperm retrieval and its subsequent use for reproduction can be challenging, particularly for illiterate or less educated individuals. Lack of awareness and education about the procedure and its ethical implications may hinder acceptance and create difficulties both during the retrieval process and after the birth of the child.

In Israel, artificial insemination using the husband’s sperm is allowed, but the use of posthumously retrieved sperm is strictly prohibited unless explicitly permitted. The Attorney General of Israel issued guidelines in 2003 that allowed post-mortem sperm retrieval and freezing based on the “presumed wishes” of the deceased (Rubinstein, 2003).

Islamic law also does not specifically address post-mortem sperm retrieval or assisted reproductive technologies (ART). In general, there is acceptance of infertility treatments, including IVF and surgical sperm retrieval methods, within the context of marriage. However, there is disagreement among Islamic scholars regarding practices such as ovum donation and surrogacy.

These varying religious perspectives highlight the complexity of ethical considerations surrounding post-mortem sperm retrieval and the need to navigate cultural and religious beliefs when addressing the practice. It is essential to engage in respectful dialogue and seek guidance from religious authorities to ensure ethical decision-making within the boundaries of religious and cultural norms. Psychological counseling of the widow and family members is an important aspect to consider before proceeding with post-mortem sperm retrieval. The process itself, as well as the potential outcomes and implications, can evoke emotional distress and psychological trauma for the bereaved family.

Open and honest communication is essential to ensure that the widow and family members have a clear understanding of the procedure and its potential outcomes. During counseling, it is important to create a safe and supportive environment where the emotional well-being of the individuals involved is prioritized. The counseling process should allow the family members to express their concerns, fears, and emotions related to post-mortem sperm retrieval.

Postmortem impregnation and the subsequent issue of parenthood raise significant medical and ethical questions. In developing nations, such as India, procedures like post-mortem sperm retrieval (PMSR) and assisted reproductive technology (ART) are still in the early stages of development. Researchers and experts are introducing new techniques to facilitate impregnation using a single healthy sperm.

The use of post-mortem sperm and the subsequent conception raise concerns about the welfare of the deceased individual, the ethical implications of using their sperm without their explicit consent, and the potential impact on any resulting offspring. The lack of specific guidelines and regulations further complicates matters, particularly when it comes to establishing the legal identity of the child, issues related to inheritance and property rights, and other legal considerations.

Murphy (1995) in a case report, highlighted an instance from Chicago in 1993 that examined the issues surrounding postmortem fatherhood in which, the transplant officer and the hospital medical director later denied the request, stating that there would still be a prima facie duty to respect any requests made by the male before brain death, and in this case, the harvesting of the sperm did not occur. This case highlights the complex ethical considerations involved in postmortem sperm retrieval and the importance of respecting the wishes expressed by individuals before brain death.

Post-mortem sperm retrieval (PMSR) raises important questions regarding the responsibility for any children that may result from the procedure. Issues such as inheritance, family relationships, and the potential social stigma and discrimination faced by children born through PMSR come into play. These children may also have concerns about their identity and heritage.

In a review paper by Tumram & Bardale (2019), the issue of parenthood after successful PMSR and assisted reproduction (AR) is explored. It is acknowledged that no child should be deprived of their father and raised solely by a single parent or grandparents. The challenges of parenting, particularly if the child is born with genetic defects or disabilities, can be more difficult for a single parent (Tumram & Bardale, 2019).

In cases where the deceased individual was never married, the responsibility for the child may fall upon the grandparents. However, the child’s existence without their biological parent raises important considerations. Can the child navigate the challenges of being raised by their grandparents? These questions are significant not only for the child but also for the family as a whole. The decision to proceed with PMSR is voluntary, and it is crucial to consider the long-term implications and potential difficulties that the child and family may face.

When considering post-mortem sperm retrieval (PMSR), it is important to take into account the medical history of the deceased individual. If the deceased person has a known severe disease condition that is likely to be passed on to future offspring and poses a serious threat to the child’s well-being, PMSR should be avoided. Comprehensive medical records and thorough screening protocols can help minimize the risk of transmitting infectious diseases during the PMSR procedure.

One of the concerns is related to privacy, as retrieving and using sperm after death raises privacy issues for the deceased and their family. There may be conflicts within families, especially if different members have differing views on the use of the deceased person’s genetic material. Some individuals may perceive the use of their genetic material without their explicit consent as a violation of their body, and it may be seen as disrespectful in Indian culture. The privacy of the deceased should be protected, and their personal information should not be disclosed or used without their consent.

## CONCLUSION

Post-mortem sperm retrieval (PMSR) is a complex and ethically challenging practice, particularly in India where there are no explicit guidelines or legislation governing its use. The loss of a loved one deeply affects families, and the desire for postmortem parenthood or the opportunity to have biological children after death is driven by various factors such as cultural beliefs, the desire for continuity, and the need for compensation for the loss of a family member.

While advancements in assisted reproductive technologies have made PMSR feasible in many countries, its implementation in India faces significant ethical, social, and cultural challenges. The lack of clear guidelines and regulations creates uncertainty and raises questions about the ethical and legal implications of the procedure.

Addressing these challenges requires spreading awareness and creating proper guidelines for PMSR. Public education and open discussions can help bridge the gap in understanding and acceptance of PMSR. It is important to consider the ethical, moral, and social risks associated with PMSR and ensure that the rights and well-being of all parties involved, including the deceased, their family, and any resulting children, are protected.

The development of comprehensive and culturally sensitive guidelines, as well as legislation, is necessary to provide a framework for the ethical practice of PMSR in India. These guidelines should consider issues of consent, privacy, dignity, autonomy, and the best interests of any resulting children.
